# CFT-Triplet: integrating contextual, frequency and textural features for fine-grained pulmonary fissure detection

**DOI:** 10.3389/frai.2026.1817367

**Published:** 2026-10-20

**Authors:** Li Xu, Yu Jiang, Rong Tao, Nanyu Li, Riqing Chen, Chenhui Su, Guodong Li, Fei Tang

**Affiliations:** 1 Shandong Public Health Clinical Center, Jinan, China; 2 Linyi People's Hospital, Linyi, China; 3 Broncus Medical Inc., Hangzhou, China; 4 Anhui Chest Hospital, Hefei, China

**Keywords:** frequency domain learning, integrity assessment, pulmonary fissure detection, state space models, triplet block

## Abstract

Accurate detection and integrity assessment of pulmonary fissures are essential for the effective treatment of emphysema and chronic obstructive pulmonary disease (COPD). However, clinical CT scans often present fissures with weak contrasts and severe deformations. Standard deep learning approaches face two fundamental limitations in this task: First, they exhibit an inherent “spectral bias,” preferentially learning low-frequency shapes while suppressing high-frequency boundary details (i.e., the fissures). Second, existing Transformer-based global modeling incurs quadratic computational costs, limiting the efficient capture of long-range anatomical continuity. To address these challenges, we propose CFT-Triplet, a framework that integrates Contextual, Frequency, and Textural features for robust fissure detection. The core of our framework is the CFT-Triplet Block, an architectural unit designed to extract and fuse these three complementary features within a unified module. Within each Triplet Block, we design a hybrid topology: (1) To model long-range anatomical dependencies, we incorporate Bidirectional Mamba2, which captures global Contextual information with linear complexity; (2) To specifically compensate for spectral loss, these contextual features are serially refined by a dedicated Frequency module, sharpening high-frequency boundary components; (3) Simultaneously, a parallel CNN-based Textural branch is utilized to extract local spatial patterns. This triplet design ensures that every network layer possesses both the global receptive field and the sensitivity to local edges. The multi-view features are then fused via a Gate-Net module to maximize complementarity. Extensive experiments on the Shandong Provincial Chest Hospital (SPCH) dataset—where 400 high-quality CT scans were selected from 500 collected cases after rigorous data cleaning (partitioned into 320 training, 40 validation, and 40 testing sets at the patient level to prevent data leakage)—demonstrate that CFT-Triplet outperforms state-of-the-art methods on our private SPCH dataset.

## 1 Introduction

Emphysema is characterized by irreversible tissue destruction and the loss of elastic recoil in the lungs, leading to a diminished quality of life. Chronic Obstructive Pulmonary Disease (COPD) is a chronic lung disease characterized by long-term airway obstruction, often accompanied by emphysema, which further leads to impaired gas exchange. As the incidence of emphysema and COPD continues to rise annually, endobronchial valves (EBV) have been shown to be an effective treatment for severe emphysema and COPD during bronchoscopic lung volume reduction (BLVR) (Koster and Slebos, 2016; Argula et al., 2013). Compared to the high risks associated with lung volume reduction surgery, EBV offers a non-invasive alternative that enhances the safety of the procedure. Prior to EBV therapy, clinicians typically perform preoperative analysis of the fissure structure to assess its completeness. In cases of incomplete fissures, adjacent lung tissues may fuse together, creating collateral ventilation pathways that can easily lead to the spread of inflammation and ipsilateral gas communication, rendering EBV treatment ineffective (Strange et al., 2007; Klooster et al., 2020). Therefore, the integrity of the fissure is a crucial criterion for selecting emphysema or COPD patients suitable for EBV therapy. Accurate fissure detection is the prerequisite for integrity assessment and a key step in supporting clinical decisions to effectively treat emphysema and COPD patients. Furthermore, fissure detection plays an important role in lesion localization and in analyzing symptoms of pulmonary diseases (Cronin et al., 2010; Group, 2001; Pan et al., 2016). Given the high prevalence of emphysema and COPD in adults, this topic is of significant clinical and societal impact.

Pulmonary fissures divide the lungs into anatomically distinct lobes. However, accurately detecting these fissures in clinical CT scans remains a significant challenge. Fissures often appear as faint, curvilinear structures with weak contrasts, and in pathological cases, they suffer from severe deformations or interruptions. Currently, CT-based fissure detection methods are generally divided into two categories: traditional methods and deep learning-based methods. Traditional methods, such as line enhancement filters (Klinder et al., 2013), ridge operators (Zhang et al., 2005), and derivative-based approaches (Wiemker et al., 2005; Xiao et al., 2016; Peng and Xiao, 2018), rely solely on hand-crafted local descriptors of fissure shape and appearance. These methods struggle to handle fissures deformed or destroyed by complex lung diseases, noise, and pathological changes. Other methods attempt to incorporate anatomical knowledge frameworks like watershed transformation (Bragman et al., 2017) or atlas-based methods (Van Rikxoort et al., 2010), but often lack generalization capabilities.

Recently, deep learning-based methods have achieved significant progress. Approaches like FissureNet (Gerard et al., 2018) and MDL-IWS (Roy et al., 2020) utilize Convolutional Neural Networks (CNNs) to extract hierarchical features. However, standard deep learning approaches face two fundamental limitations in this specific task:

First, standard deep learning approaches are fundamentally constrained by the “Spectral Bias” problem (Rahaman et al., 2019). Theoretical analysis reveals that neural networks exhibit an inherent tendency to prioritize the convergence of low-frequency components (e.g., global organ shapes) while suppressing high-frequency details (Rahaman et al., 2019). This limitation is not exclusive to CNNs but is also prevalent in Vision Transformers (ViTs). Recent Fourier domain studies demonstrate that the Multi-Head Self-Attention (MSA) mechanism functions as a spatial low-pass filter (Park and Kim, 2022), which attenuates high-frequency signals and leads to the “oversmoothing” phenomenon in deeper layers (Wang et al., 2022). Furthermore, unlike CNNs that capture local edges in early layers, ViTs tend to aggregate global information prematurely, potentially sacrificing local high-frequency precision (Raghu et al., 2021). Consequently, pulmonary fissures, which represent extremely high-frequency spatial discontinuities (edges), are prone to being filtered out or blurred during the feature extraction process. Existing methods typically rely on single-domain spatial descriptors and fail to explicitly compensate for this spectral information loss.

Second, the Computational Bottleneck. While Transformer-based models have been introduced to capture global anatomical dependencies, their self-attention mechanism incurs quadratic computational costs (*O*(*N*^2^)). This limits their ability to process high-resolution 3D CT volumes efficiently, often forcing a trade-off between input resolution and the receptive field, which disrupts the continuity of long-range fissure structures.

To address these challenges, we propose CFT-Triplet, a framework that integrates Contextual, Frequency, and Textural features for robust pulmonary fissure detection. Unlike previous methods that simply stack CNNs and Transformers, we introduce a unified CFT-Triplet Block designed to extract and fuse these three complementary features. Specifically, we employ Bidirectional Mamba2, a State Space Model (SSM), to capture global contextual information with linear complexity, overcoming the efficiency bottleneck of Transformers. Furthermore, to mitigate spectral bias, we design a dedicated Frequency module that serially refines the contextual features, explicitly sharpening high-frequency boundary components. Simultaneously, a parallel CNN branch captures local textural patterns. Extensive experiments on our collected Shandong Provincial Chest Hospital (SPCH) dataset demonstrate the performance of our method.

In summary, the main contributions of this work are as follows:

- We propose an architecture centered on the CFT-Triplet Block, which integrates Contextual, Frequency, and Textural features. This hybrid topology ensures that every network layer possesses both the global receptive field and the sensitivity to local edges, improving detection specificity in noisy or low-contrast regions.
- We incorporate Bidirectional Mamba2 to model long-range anatomical dependencies. Unlike traditional Transformers, Mamba2 captures global context with linear computational complexity, allowing for processing of 3D CT scans without sacrificing resolution or anatomical continuity.
- We introduce a dedicated Frequency module to explicitly address the inherent spectral bias of deep networks. By serially refining contextual features in the frequency domain, this module sharpens the high-frequency components of weak boundaries, facilitating the precise detection of “invisible” or abnormal fissures.
- To address the data scarcity issue, we constructed the SPCH dataset, comprising 400 high-quality 3D CT scans selected from 500 clinical cases after stringent data screening. This dataset covers a wide range of COPD severities and serves as a benchmark for fissure detection tasks.

The remainder of this paper is organized as follows. Section 2 details the architecture of the proposed detection network. Section 3 presents the experimental results and comparisons. Section 4 concludes the study.

## 2 CFT-Triplet

### 2.1 CFT-Triplet framework

The detailed architectural configuration of the proposed CFT-Triplet is presented in Table 1, and the overall workflow is illustrated in Figure 1. Our framework is constructed by stacking the proposed Triplet Blocks and Fusion Layers within a hierarchical Encoder-Decoder structure.

**Table 1:** Detailed architectural configuration of the proposed CFT-Triplet framework.

Layer	Operations	Output size
Encoder		
Conv	Conv 3 × 3 × 3, *C* = 5, *S* = 2, *P* = 1	1 × 5 × 160 × 160 × 160
Conv block	Conv 3 × 3 × 3, *C* = 48, *S* = 2, *P* = 1 Conv 3 × 3 × 3, *C* = 48, *S* = 1, *P* = 1 Conv 3 × 3 × 3, *C* = 48, *S* = 1, *P* = 1	1 × 48 × 80 × 80 × 80
DownSampling	Conv 3 × 3 × 3, *C* = 96, *S* = 2, *P* = 1	1 × 96 × 40 × 40 × 40
Triplet block	Textural branch + Contextual branch + Frequency branch	1 × 96 × 40 × 40 × 40
Fusion layer	1 Gate-Net + 3 Conv 3 × 3 × 3	1 × 96 × 40 × 40 × 40
DownSampling	Conv 3 × 3 × 3, *C* = 192, *S* = 2, *P* = 1	1 × 192 × 20 × 20 × 20
Triplet block	Textural branch + Contextual branch + Frequency branch	1 × 192 × 20 × 20 × 20
Fusion layer	1 Gate-Net + 3 Conv 3 × 3 × 3	1 × 192 × 20 × 20 × 20
DownSampling	Conv 3 × 3 × 3, *C* = 384, *S* = 2, *P* = 1	1 × 384 × 10 × 10 × 10
Triplet block	Textural branch + Contextual branch + Frequency branch	1 × 384 × 10 × 10 × 10
Fusion layer	1 Gate-Net + 3 Conv 3 × 3 × 3	1 × 384 × 10 × 10 × 10
DownSampling	Conv 3 × 3 × 3, *C* = 768, *S* = 2, *P* = 1	1 × 768 × 5 × 5 × 5
Bottleneck		
Bottleneck	Conv 3 × 3 × 3, *C* = 768, *S* = 1, *P* = 1 Conv 3 × 3 × 3, *C* = 768, *S* = 1, *P* = 1 Conv 3 × 3 × 3, *C* = 768, *S* = 1, *P* = 1	1 × 768 × 5 × 5 × 5
Decoder		
UpSampling	ConvTranspose3D 2 × 2 × 2, *C* = 384, *S* = 2	1 × 384 × 10 × 10 × 10
Fusion Layer	1 Gate-Net + 3 Conv 3 × 3 × 3	1 × 384 × 10 × 10 × 10
UpSampling	ConvTranspose3D 2 × 2 × 2, *C* = 192, *S* = 2	1 × 192 × 20 × 20 × 20
UpSampling	ConvTranspose3D 2 × 2 × 2, *C* = 96, *S* = 2	1 × 96 × 40 × 40 × 40
UpSampling	ConvTranspose3D 2 × 2 × 2, *C* = 48, *S* = 2	1 × 48 × 80 × 80 × 80
Triplet block	Textural branch + Contextual branch + Frequency branch	1 × 48 × 80 × 80 × 80
Fusion layer	1 Gate-Net + 3 Conv 3 × 3 × 3	1 × 48 × 80 × 80 × 80
UpSampling	ConvTranspose3D 2 × 2 × 2, *C* = 48, *S* = 2	1 × 48 × 160 × 160 × 160
Triplet block	Textural branch + Contextual branch + Frequency branch	1 × 48 × 160 × 160 × 160
Fusion layer	1 Gate-Net + 3 Conv 3 × 3 × 3	1 × 48 × 160 × 160 × 160
ConvHead	Conv 3 × 3 × 3, *C* = 64, *S* = 1, *P* = 1 Conv 3 × 3 × 3, *C* = 32, *S* = 1, *P* = 1 Conv 3 × 3 × 3, *C* = 2, *S* = 1, *P* = 1	1 × 2 × 160 × 160 × 160
MoE-fusion	Upsample(*P*_1~4_)+Concat+GAP+MLP → weighted sum	1 × 2 × 160 × 160 × 160

**Figure 1:**
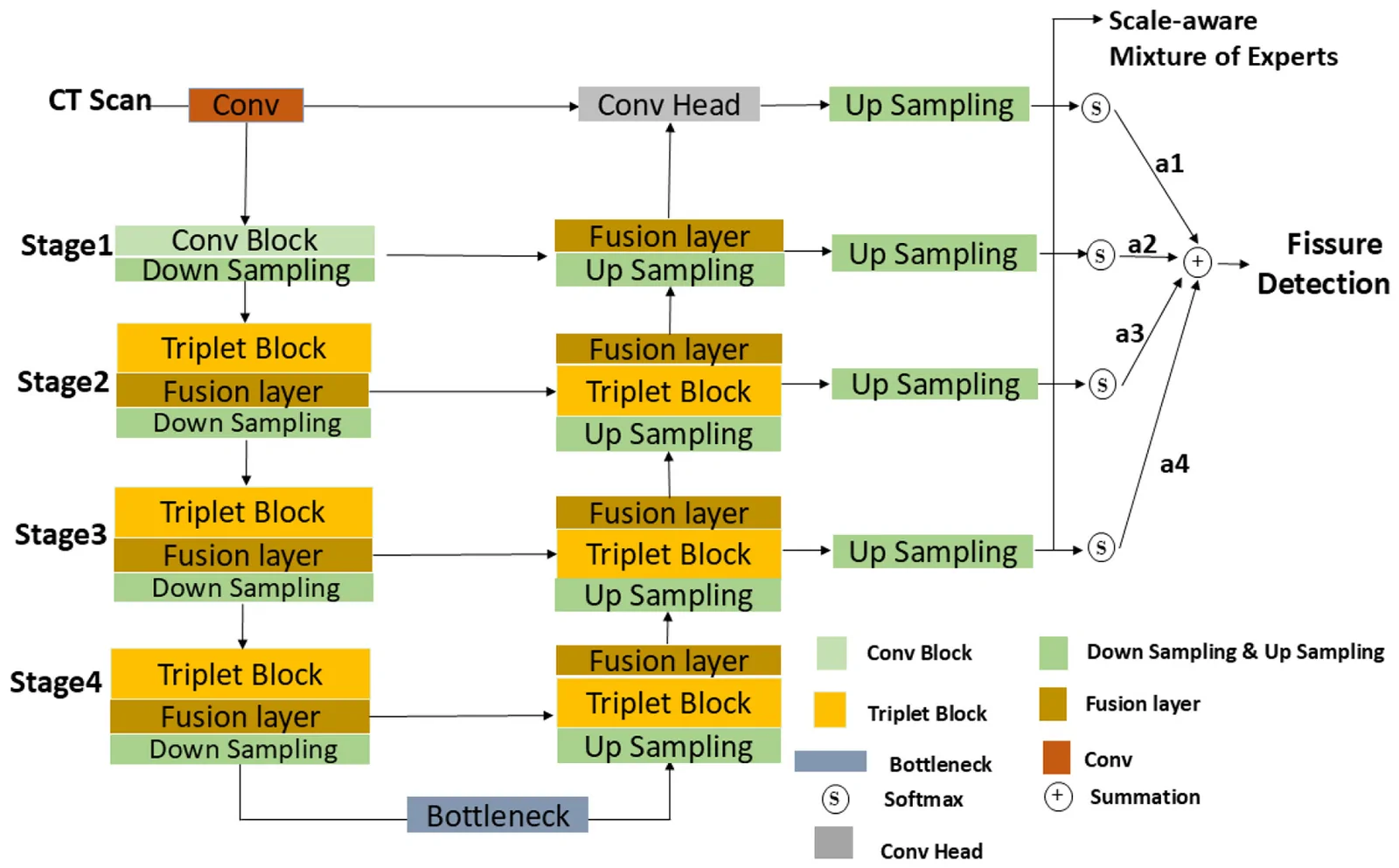
An overview of the proposed fissure detection network (CFT-Triplet).

The hierarchical encoder accepts a multi-channel input (CT volume fused with distance and orientation maps) of size 160 × 160 × 160. Rather than using standard pooling, we employ strided convolutions (*S* = 2) in the DownSampling layers to progressively reduce the spatial resolution from 160^3^ to 5^3^ across five stages. Correspondingly, the feature channels are expanded stage-by-stage (48 → 96 → 192 → 384 → 768) to enrich the semantic representation. Crucially, after each downsampling operation, the feature maps are processed by the Triplet Block. As detailed in Section 2.4, this block simultaneously models local textures (CNN), global context (Mamba2), and spectral boundaries (Frequency) in parallel. The outputs are then integrated via a Fusion Layer containing Squeeze-and-Excitation (SE) modules, ensuring that informative features are adaptively recalibrated before propagating to the next stage.

The bottleneck stage operates at the lowest resolution (5 × 5 × 5) with the highest channel dimension (*C* = 768). It comprises a stack of three convolutional layers designed to consolidate the most abstract semantic information, serving as a robust bridge between the contracting and expanding paths.

The reconstruction decoder aims to restore the spatial details of the fissures. We utilize UpSampling layers (Transpose Convolutions) to progressively upscale the feature maps. To mitigate the loss of high-frequency boundary information caused by downsampling, we employ skip connections that concatenate the upsampled features with their high-resolution counterparts from the encoder. To ensure symmetric feature learning, our decoder also employs the Triplet Block and Fusion Layer at each stage. This design ensures that the restoration process is guided not only by spatial information but also by global anatomical constraints and frequency-domain boundary sharpening. Finally, the detection Head projects the 48-channel high-resolution features into a probability map using a sequence of 1 × 1 × 1 convolutions, culminating in a 2-channel output for fissure detection.

### 2.2 Encoder

To extract discriminative feature representations from CT images, the encoder is constructed by stacking the proposed CFT-Triplet Blocks in a hierarchical manner. As the core feature extraction unit, the Triplet Block employs a hybrid architecture comprising three complementary branches to model the complex anatomy of pulmonary fissures. Specifically, the Textural Branch utilizes cascaded convolutional layers to capture local spatial patterns and high-frequency edge details independent of global noise. In parallel, the Contextual Branch incorporates the Bidirectional Mamba2 model to establish long-range anatomical dependencies with linear computational complexity, effectively encoding the global shape continuity. To address the spectral bias inherent in deep networks, a dedicated Frequency Branch is arranged in series to refine these contextual features via spectral sharpening in the frequency domain. These multi-view features are subsequently integrated through a Gate-Net mechanism within the Fusion Layer. By progressively applying this triplet extraction process across downsampled resolutions, the encoder effectively generates high-level semantic representations that preserve both the global structural continuity and the precise local boundaries of the fissures.

### 2.3 Decoder

As detailed in Table 1 and illustrated in Figure 1, the decoder follows a symmetric hierarchical design to the encoder, progressively restoring the spatial resolution from 5 × 5 × 5 to 160 × 160 × 160. To effectively handle the topological variability of fissures, we incorporate a Scale-aware Mixture of Experts (MoE) mechanism into the decoding process.

The decoding process proceeds through five upsampling stages. In each stage, feature maps are upsampled via Transposed Convolutions and concatenated with the corresponding high-resolution features from the encoder. To ensure symmetric feature learning, we deploy the proposed CFT-Triplet Block and Fusion Layer at each stage. This design allows the network to continuously refine global anatomical context (via Mamba2) and sharpen high-frequency boundaries (via Frequency Branch) during the restoration process.

Instead of relying on a single output from the final layer, we treat the intermediate prediction heads from the first four decoding stages as Scale-Specific Experts (Chen et al., 2022). As listed in Table 1, we extract four auxiliary predictions (*P*_1_, *P*_2_, *P*_3_, *P*_4_) with resolutions ranging from 10^3^ to 80^3^. These experts provide diverse views of the detection task: low-resolution experts (*P*_1_, *P*_2_) excel at capturing coarse-grained global shapes, while high-resolution experts (*P*_3_, *P*_4_) focus on fine-grained local boundary details.

Unlike traditional deep supervision strategies that rely on static learnable parameters, our MoE module employs a dynamic gating network to generate instance-specific weights. The fusion process consists of three steps: (1) **Aggregation:** Since the expert predictions (*P*_1_ to *P*_4_) have different spatial resolutions, they are first bilinearly upsampled to the target output size (160 × 160 × 160) and then concatenated along the channel dimension to form a multi-scale feature bank. (2) **Gating:** A Global Average Pooling (GAP) layer compresses the aggregated features into a global context vector. This vector is then mapped by a lightweight Multi-Layer Perceptron (MLP) and normalized by a Softmax function to produce dynamic weights *w*_*i*_(*x*) (where $\overset{4}{\underset{i=1}{\sum }}w_{i}=1$). (3) **Fusion:** To ensure that the fusion operates on calibrated probabilities rather than raw logits, the upsampled expert predictions are normalized via a softmax function. The final detection output is obtained by the weighted summation (Equation 1):


$$\begin{array}{l} P_{out}=\overset{4}{\underset{i=1}{\sum }}w_{i}\left(x\right)\cdot \text{Softmax}\left(\text{Upsample}\left(P_{i}\right)\right) \end{array}$$ (1)

This mechanism allows CFT-Triplet to adaptively emphasize specific scales based on the instance-level complexity, improving robustness against complex anatomical variations.

### 2.4 CFT-Triplet block components

Unlike standard multi-branch blocks that operate purely in parallel, the proposed CFT-Triplet Block adopts a hybrid parallel-series architecture to integrate textural, contextual, and spectral information. As illustrated in Figure 1, the block consists of two primary parallel streams: the Texture Branch and the Contextual-Frequency Branch. Notably, within the latter stream, the Contextual Branch and the Frequency Branch are arranged in series. This specific design allows for a progressive refinement of features, where contextual representations are modulated by spectral analysis, while the parallel Texture Branch independently preserves fine-grained spatial details.

#### 2.4.1 Texture branch

To mitigate the loss of local pixel-level relations, the Textural Branch processes the input *X*_*in*_ independently to extract high-frequency local details. As illustrated in the top stream of Figure 1, this branch comprises two cascaded residual blocks. Each block sequentially employs a standard convolution and a depthwise convolution with 3 × 3 × 3 kernels to refine local features (Equation 2).


$$\begin{array}{l} \begin{array}{l} B\left(X_{in}\right)=x+\psi \left(\text{DWConv}\left(\psi \left(\text{Conv}\left(X_{in}\right)\right)\right)\right)\\ \;X_{T}=B\left(B\left(X_{in}\right)\right) \end{array} \end{array}$$ (2)

where Conv and DWConv denote 3 × 3 × 3 standard and depthwise convolutions, respectively, and ψ represents the composite function of Batch Normalization (BN) and GELU activation.

#### 2.4.2 Contextual branch

To capture long-range anatomical dependencies efficiently, the first stage of the cascaded stream constitutes the Contextual Branch. As illustrated in the bottom stream of Figure 1, this branch employs a bidirectional modeling strategy integrating the Mamba2 architecture. Unlike traditional SSMs, Mamba2 introduces the State Space Duality (SSD) framework (Dao and Gu, 2024; Shi et al., 2025), which enables efficient parallel training via matrix multiplication. The input feature *X*_*in*_ is first normalized via Layer Normalization (LN) and then processed through two parallel paths. We define two sequence transformation operators, $T_{fwd}\left(\cdot \right)$ and $T_{bwd}\left(\cdot \right)$, which flatten and arrange the visual features into causal (start-to-end) and anti-causal (end-to-start) sequences, respectively. Specifically, in our implementation, the Mamba2 module is configured with an expansion factor *E* = 2, a state dimension *D*_*state*_ = 16, and a 3D depthwise convolution kernel size of 3 × 3 × 3. The outputs from the causal and anti-causal paths are combined via element-wise addition to form a unified bidirectional representation before being passed to the Frequency Branch. Formally, the dual-path processing is formulated as Equation 3:


$$\begin{array}{l} \begin{array}{c} X_{fwd}=X_{in}+\text{Mamba2}\left(T_{fwd}\left(\text{LN}\left(X_{in}\right)\right)\right),\\ X_{bwd}=X_{in}+\text{Mamba2}\left(T_{bwd}\left(\text{LN}\left(X_{in}\right)\right)\right), \end{array} \end{array}$$ (3)

where Mamba2(·) denotes the core mixing block governed by the SSD framework. $T_{fwd}$ represents the standard flattening operator, while $T_{bwd}$ reverses the sequence order to capture bidirectional dependencies. The resulting features *X*_*fwd*_ and *X*_*bwd*_ are subsequently fused to generate the global context feature *X*_*C*_.

#### 2.4.3 Frequency branch

To address the potential boundary blurring in global features, the Frequency Branch is arranged in **series** following the Contextual Branch. Instead of processing the raw input, it takes the outputs from the Mamba2 modules (*X*_*fwd*_ and *X*_*bwd*_) as inputs. This cascaded design allows the network to explicitly sharpen the semantic features extracted by Mamba in the spectral domain. As shown in Figure 1, the branch maintains the dual-path structure, where each path consists of a Weighted FFT block for spectral filtering and an MLP block for channel mixing. For a given input feature *x* (representing either *X*_*fwd*_ or *X*_*bwd*_), the process is defined as Equation 4:


$$\begin{array}{l} \begin{array}{c} x_{freq}=x+\text{IFFT}\left(W⊙\text{FFT}\left(\text{LN}\left(x\right)\right)\right),\\ x_{out}=x_{freq}+\text{MLP}\left(\text{LN}\left(x_{freq}\right)\right), \end{array} \end{array}$$ (4)

where *W* is a learnable parameter in the frequency domain, and ⊙ denotes element-wise multiplication. Finally, the refined forward and backward features are adaptively fused via a learnable gating parameter *p* to produce the combined context-frequency feature *X*_*CF*_ (Equation 5):


$$\begin{array}{l} X_{CF}=x_{out}^{fwd}\cdot p^{-1}+x_{out}^{bwd}\cdot p \end{array}$$ (5)

where $x_{out}^{fwd}$ and $x_{out}^{bwd}$ denote the outputs processed from the forward and backward paths, respectively. Here, *p*∈ℝ^*C*^ is a learnable channel-wise vector rather than a simple scalar or spatial tensor. This channel-wise gating mechanism acts as an efficient channel attention strategy, dynamically balancing the forward and backward spectral features without the quadratic computational overhead of full spatial self-attention. Unlike GFNet (Rao et al., 2023), the necessity of our serial design lies in explicitly compensating for the inherent high-frequency loss (spectral bias) of Mamba2, thereby sharpening fuzzy boundaries within the established global context.

### 2.5 Fusion layer

As illustrated in Figure 2, the Fusion Layer is designed to adaptively integrate the complementary information from the two parallel streams. For notational brevity, we denote the output features from the Local Texture Branch as *X*_*T*_ (representing textural details) and the features from the Cascaded Context & Frequency Stream as *X*_*CF*_ (representing sharpened global context).

**Figure 2:**
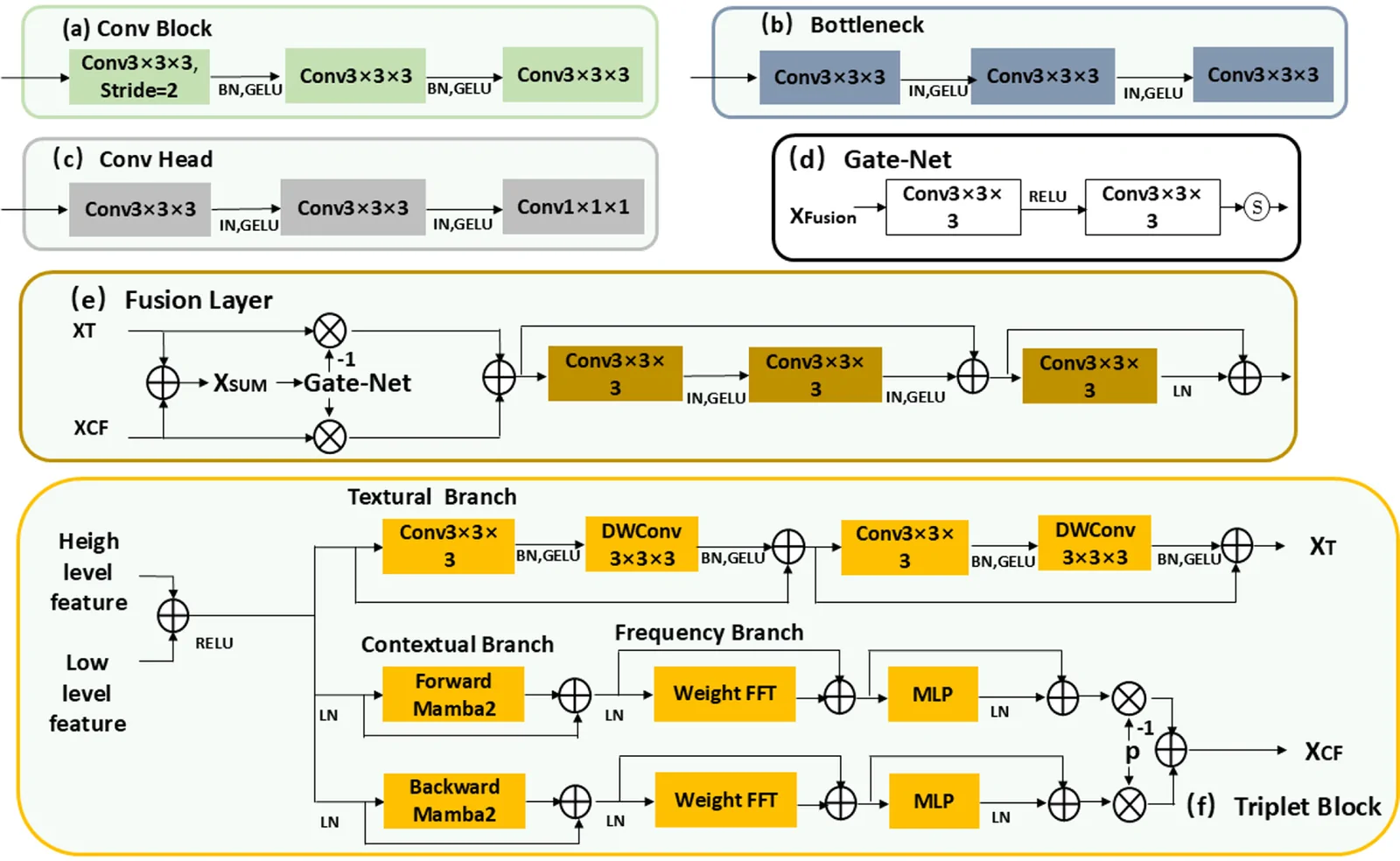
The schematic illustration of the Conv Block, Bottleneck, Conv Head, Fusion Layer and Triplet Block architecture.

To effectively modulate these distinct representations, we employ a Gate-Net mechanism. First, the features from both branches are summed to aggregate the initial information (Equation 6):


$$\begin{array}{l} X_{Sum}=X_{T}+X_{CF} \end{array}$$ (6)

Next, the aggregated feature *X*_*Sum*_ is passed through the Gate-Net to generate a spatial attention map. The Gate-Net is composed of two 3 × 3 × 3 convolutional layers, a ReLU activation, and a Sigmoid activation, ensuring the output weights *g* are in the range (0, 1) (Equation 7):


$$\begin{array}{l} g=\sigma \left(\text{GateNet}\left(X_{Sum}\right)\right) \end{array}$$ (7)

Finally, we perform a weighted fusion based on the learned gate *g*. This allows the network to dynamically balance the trade-off between global semantics and local boundaries (Equation 8):


$$\begin{array}{l} X_{Fused}=g⊙X_{CF}+\left(1-g\right)⊙X_{T} \end{array}$$ (8)

where ⊙ denotes element-wise multiplication. By this design, *X*_*CF*_ contributes global structural consistency in homogeneous regions, while *X*_*T*_ preserves high-frequency details at object boundaries. Following the weighted fusion, the feature map *X*_*Fused*_ is further processed by a residual block consisting of three 3 × 3 × 3 convolutional layers with Instance Normalization (IN) and GELU activation to ensure smooth feature integration.

### 2.6 Training loss

To address the severe class imbalance inherent in pulmonary fissure detection (where fissures occupy only a tiny fraction of the volume), we employ a hybrid loss function combining the Tversky Loss (Salehi et al., 2017) and the Weighted Binary Cross Entropy (WBCE) loss (De Boer et al., 2005). The Tversky Loss is adopted to balance the precision and recall, allowing us to penalize false negatives (FNs) more heavily than false positives (FPs). It is defined as Equation 9:


$$\begin{array}{l} TverskyLoss\left(\alpha ,\beta \right)=\frac{\sum_{i=1}^{N}p_{1i}g_{1i}}{\sum_{i=1}^{N}p_{1i}g_{1i}+\alpha \sum_{i=1}^{N}p_{1i}g_{0i}+\beta \sum_{i=1}^{N}p_{0i}g_{1i}} \end{array}$$ (9)

where *p*_1*i*_ and *g*_1*i*_ denote the predicted probability and ground truth for the fissure class, respectively. The hyperparameters α and β control the trade-off between FPs and FNs. In our experiments, we set α = 0.3 and β = 0.7 to emphasize the recall of subtle fissures.

Additionally, the Weighted BCE Loss provides stable gradient feedback for pixel-level classification (Equation 10):


$$\begin{array}{l} WBCELoss=-w\cdot \left[y\log\left(p\right)+\left(1-y\right)\log\left(1-p\right)\right] \end{array}$$ (10)

where *w* is a class weighting factor set to 100 to mitigate the dominance of background voxels. The total objective function is the weighted sum (Equation 11):


$$\begin{array}{l} TotalLoss=TverskyLoss+\lambda \times WBCELoss \end{array}$$ (11)

where λ is empirically set to 1. Note that this loss is applied to the final output *P*_*out*_ generated by the MoE fusion module.

## 3 Experiments

### 3.1 Dataset and ethics

The SPCH dataset used in this study was retrospectively collected from Shandong Provincial Chest Hospital. This study was conducted in accordance with the Declaration of Helsinki and was approved by the Institutional Review Board (IRB) of the hospital (Approval No. IRB-2025-089). Initially, a total of 500 chest CT scans were retrieved. To ensure high data quality, a stringent data cleaning and screening process was implemented. After excluding low-quality samples, 400 valid scans were selected and partitioned into training (320 cases), validation (40 cases), and testing (40 cases) subsets at the patient level to prevent data leakage. The dataset includes scans from multiple vendors (Siemens, Philips, GE Medical Systems) with diverse imaging protocols (0.625–1.25 mm slice thickness, 100–120 kVp), introducing natural variability to evaluate model generalizability. The specific details of data acquisition and processing are as follows:

1. Data collection and cleaning: The dataset covers the period from June 2014 to November 2020. We applied strict exclusion criteria during the data cleaning phase. Scans were excluded if they exhibited: (i) significant motion or metallic artifacts that degraded image quality; (ii) incomplete coverage of the lung field; (iii) non-standard slice thickness or low resolution; or (iv) severe pathologies (e.g., massive effusion or large tumors) that completely obliterated the anatomical structure of the lung fissures. Consequently, 100 unsuitable cases were removed from the initial cohort.
2. Informed consent: The data collection followed a standard subject recruitment protocol. Informed consent was obtained from all participating patients (or their legal guardians) included in this study.
3. Ground truth annotation: To establish the gold standard for segmentation, two experienced clinical radiologists independently annotated the pulmonary fissures at the voxel level. These initial annotations were then subjected to a double-blind review and final verification by a senior specialist with a title of Associate Chief Physician or higher to resolve any discrepancies.
4. Algorithm development: The deep learning-based lung fissure detection network was developed and optimized by two AI algorithm engineers, who conducted the training and testing procedures on the prepared dataset.
5. Clinical validation: To ensure clinical relevance, the final detection results generated by the network were reviewed by two medical professionals (Associate Chief Physician level or higher). They provided qualitative assessment, while the AI engineers computed precision evaluation metrics.

### 3.2 Experimental details

The proposed CFT-Triplet framework was implemented using the PyTorch library and accelerated on NVIDIA A100 GPUs. To standardize the heterogeneous raw data, we adopted the self-configuring preprocessing pipeline from the nnFormer framework (Zhou et al., 2023). Distinct from manual windowing strategies, we employed a heuristic-based intensity normalization: voxel values were clipped to the 0.5th and 99.5th percentiles of the foreground to eliminate outliers, followed by z-score normalization (subtracting the mean and dividing by the standard deviation). Spatially, the volumes were cropped to the region of non-zero values to reduce computational overhead, and subsequently resampled to the median spacing of the dataset using third-order spline interpolation to ensure isotropic resolution.

Given the high memory requirements of 3D volumetric processing, the network was trained on sub-volume patches of size 160 × 160 × 160 with a batch size of 8. We employed a data augmentation strategy—including random horizontal flipping, spatial shifting, Gaussian smoothing (σ = 1), and intensity jittering—to enhance the model's generalization capability. Optimization was performed using the AdamW optimizer (Kingma and Ba, 2014) initialized with a learning rate of 1 × 10^−4^, which was dynamically adjusted via a supervised decay strategy (coefficient 0.3). For inference, the final detection masks were generated by applying a threshold of 0.5 to the predicted probability maps. Detailed hyperparameters and tuning protocols for all baseline models are provided in Supplementary Table S1.

### 3.3 Comparative models and metrics

#### 3.3.1 Baseline comparisons

To evaluate the proposed CFT-Triplet, we benchmarked it against several detection approaches. The comparison group covers four architectural paradigms:

- *CNN-based methods:* We selected the widely-used U-Net (Ronneberger et al., 2015), V-Net (Milletari et al., 2016), and Attention U-Net (Oktay et al., 2018) as standard baselines.
- *Transformer-based methods:* To represent attention mechanisms, we included UNETR (Chen et al., 2020), TransBTS (Wang et al., 2021), Swin UNETR (Hatamizadeh et al., 2021), and nnFormer (Zhou et al., 2023). Note that these methods are hybrid architectures leveraging both CNNs for local feature extraction and Transformers for global context modeling.
- *Frequency-based methods:* We incorporated GFNet (Rao et al., 2023), which utilizes Fast Fourier Transform (FFT) for global token mixing. This serves as a direct baseline to evaluate our learnable Frequency Branch.
- *State space models (Mamba-based):* To evaluate the efficacy of SSMs, we compared against U-Mamba (Ma et al., 2024) and SegMamba-V2 (Xing et al., 2025). These models adapt the Mamba architecture for 3D volumetric medical detection.

For an unbiased assessment, all competing models were retrained on our dataset using identical data splits, loss functions, and hardware specifications.

#### 3.3.2 Evaluation metrics

Given the extremely thin and continuous anatomical nature of pulmonary fissures, standard pixel-wise metrics often fail to capture clinically significant errors such as boundary misalignment and topological breaks. Therefore, we adopted a multi-dimensional evaluation protocol comprising four metrics:

*1) Volumetric overlap & precision:* We employed the Dice Similarity Coefficient (DSC) and Precision (Pre) to measure the voxel-level classification accuracy (Equation 12).


$$\begin{array}{l} DSC=\frac{2|A\cap B|}{\left|A|+|B\right|},\;Pre=\frac{TP}{TP+FP} \end{array}$$ (12)

*2) Boundary adherence (thin structure):* Since fissures are sheet-like structures with 1-2 pixel width, we utilized the Normalized Surface Dice (NSD). Unlike standard DSC, NSD measures the overlap of surfaces within a specified tolerance τ (set to 1mm), providing an assessment for thin anatomical boundaries (Equation 13).


$$\begin{array}{l} NSD=\frac{\left|S_{A}^{\left(\tau \right)}\cap S_{B}|+|S_{A}\cap S_{B}^{\left(\tau \right)}\right|}{\left|S_{A}|+|S_{B}\right|} \end{array}$$ (13)

where *S*^(τ)^ denotes the surface region within tolerance τ.

*3) Topological continuity:* To evaluate fissure integrity (i.e., whether the fissure is continuous or broken), we calculated the Betti Number Error (BNE). Betti numbers (β_0_, β_1_, β_2_) characterize the topology of objects (e.g., connected components, holes). BNE measures the absolute difference between the topology of the prediction and the ground truth (Equation 14).


$$\begin{array}{l} BNE=|\beta_{0}\left(A\right)-\beta_{0}\left(B\right)|+|\beta_{1}\left(A\right)-\beta_{1}\left(B\right)| \end{array}$$ (14)

Lower BNE indicates better topological preservation (fewer broken fissures). Clinically, fissure integrity is critical for determining collateral ventilation before endobronchial valve (EBV) therapy. Lower BNE directly correlates with fewer artificial breaks or holes in the predicted fissures, aligning with the physician's visual assessment of fissure completeness. In our implementation, BNE was computed using the *Gudhi* topological data analysis library based on cubical complexes, after binarizing the probability maps with a threshold of 0.5.

### 3.4 Comparison results

Table 2 presents the quantitative comparison results against state-of-the-art medical detection methods. The last column reports p-values from a Wilcoxon signed-rank test on DSC. CFT-Triplet significantly outperforms all compared methods (*p* < 0.05), with the improvement over the strongest baseline SegMamba-V2 (88.62% vs. 85.58%) yielding *p* = 0.0083.

**Table 2:** Quantitative performance comparisons on the testing dataset.

Type	Methods	DSC (%) ↑	Pre (%) ↑	NSD (%) ↑	BNE ↓	*p*-value
CNN	UNet (Ronneberger et al., 2015)	79.68 ± 4.15	80.90 ± 3.80	81.50 ± 4.50	1.720 ± 0.950	0.00012
	VNet (Milletari et al., 2016)	78.85 ± 3.30	81.01 ± 3.50	80.25 ± 4.20	1.850 ± 1.020	0.00027
	Atten Unet (Oktay et al., 2018)	81.15 ± 2.65	80.80 ± 2.85	83.40 ± 3.10	1.450 ± 0.750	0.0043
Transformer	UNETR (Chen et al., 2020)	74.83 ± 5.27	78.09 ± 5.41	76.20 ± 5.80	1.350 ± 0.880	0.00098
	TransBTS (Wang et al., 2021)	79.34 ± 2.55	79.41 ± 2.95	81.10 ± 3.25	1.120 ± 0.650	0.00061
	Swin UNETR (Hatamizadeh et al., 2021)	82.58 ± 4.12	81.47 ± 4.01	85.30 ± 3.50	0.950 ± 0.550	0.0016
	nnFormer (Zhou et al., 2023)	83.92 ± 3.85	83.65 ± 3.50	86.85 ± 3.20	0.820 ± 0.480	0.0037
Frequency	GFNet (Rao et al., 2023)	83.15 ± 3.55	82.26 ± 3.45	86.10 ± 3.60	0.920 ± 0.520	0.0019
Mamba	U-Mamba (Ma et al., 2024)	84.92 ± 2.30	84.34 ± 2.25	88.40 ± 2.50	0.450 ± 0.250	0.0045
	SegMamba-V2 (Xing et al., 2025)	85.58 ± 2.35	84.78 ± 2.40	89.50 ± 2.20	0.320 ± 0.180	0.0083
**Ours**	**CFT-Triplet**	88.62 ± 2.19	88.18 ± 2.05	92.15 ± 1.85	0.300 ± 0.150	–

NSD, Normalized surface dice (evaluates boundary precision); BNE, Betti number error (evaluates topological connectivity, lower is better). The best results are highlighted in bold. A paired Wilcoxon signed-rank test confirmed that CFT-Triplet's DSC improvements are statistically significant over all compared methods (all *p* < 0.05).

Classic CNN-based models (UNet, VNet) achieve reasonable voxel-level overlap (DSC ≈ 79%–80%). However, due to their inherently local receptive fields, they struggle to capture the global continuity of curvilinear structures. This limitation is reflected in their high BNE scores (≈ 1.7–1.8), indicating that fissures are frequently predicted as disconnected fragments.

In contrast, Transformer-based methods (e.g., UNETR, nnFormer) leverage self-attention mechanisms to model long-range dependencies. Consequently, they exhibit improved topological preservation compared to CNNs, reducing the BNE to approximately 0.8–1.3. Nevertheless, they still face challenges in delineating the precise boundaries of extremely thin structures, as shown by the NSD metrics.

The Mamba-based methods, SegMamba-V2 and U-Mamba, further enhance connectivity (BNE ≈ 0.3–0.4) by utilizing linear complexity sequence modeling. Building upon these advancements, CFT-Triplet records the lowest BNE of 0.300, surpassing the runner-up SegMamba-V2 (0.320). While Mamba-based methods generally ensure good connectivity, our model's performance in NSD (92.15%) and DSC (88.62%) indicates that the addition of the Frequency Branch sharpens boundaries without compromising topological integrity. The qualitative visual comparisons on two typical clinical cases are illustrated in Figures 3, 4.

**Figure 3:**
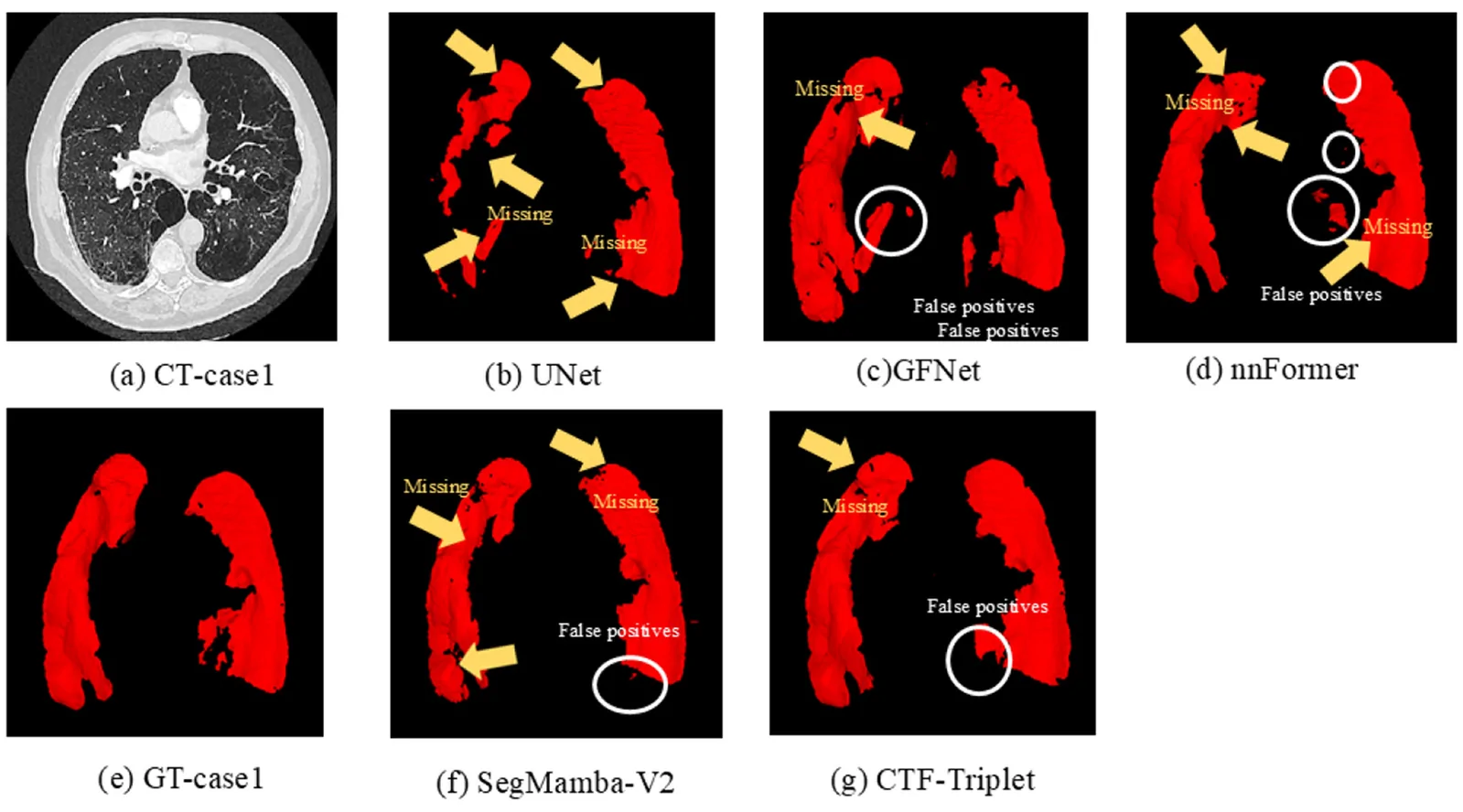
Visual comparisons of detection results generated by different models. **(a)** Coronal plane of the original 3D CT-1. **(b)** Ground truth (manual annotation). **(b–f)** Visualization of results from comparison models (UNet, GFNet, nnFormer, GT-case1, SegMamba-V2). **(g)** Detection result of the proposed CFT-Triplet model. The 3D views are provided for each result.

**Figure 4:**
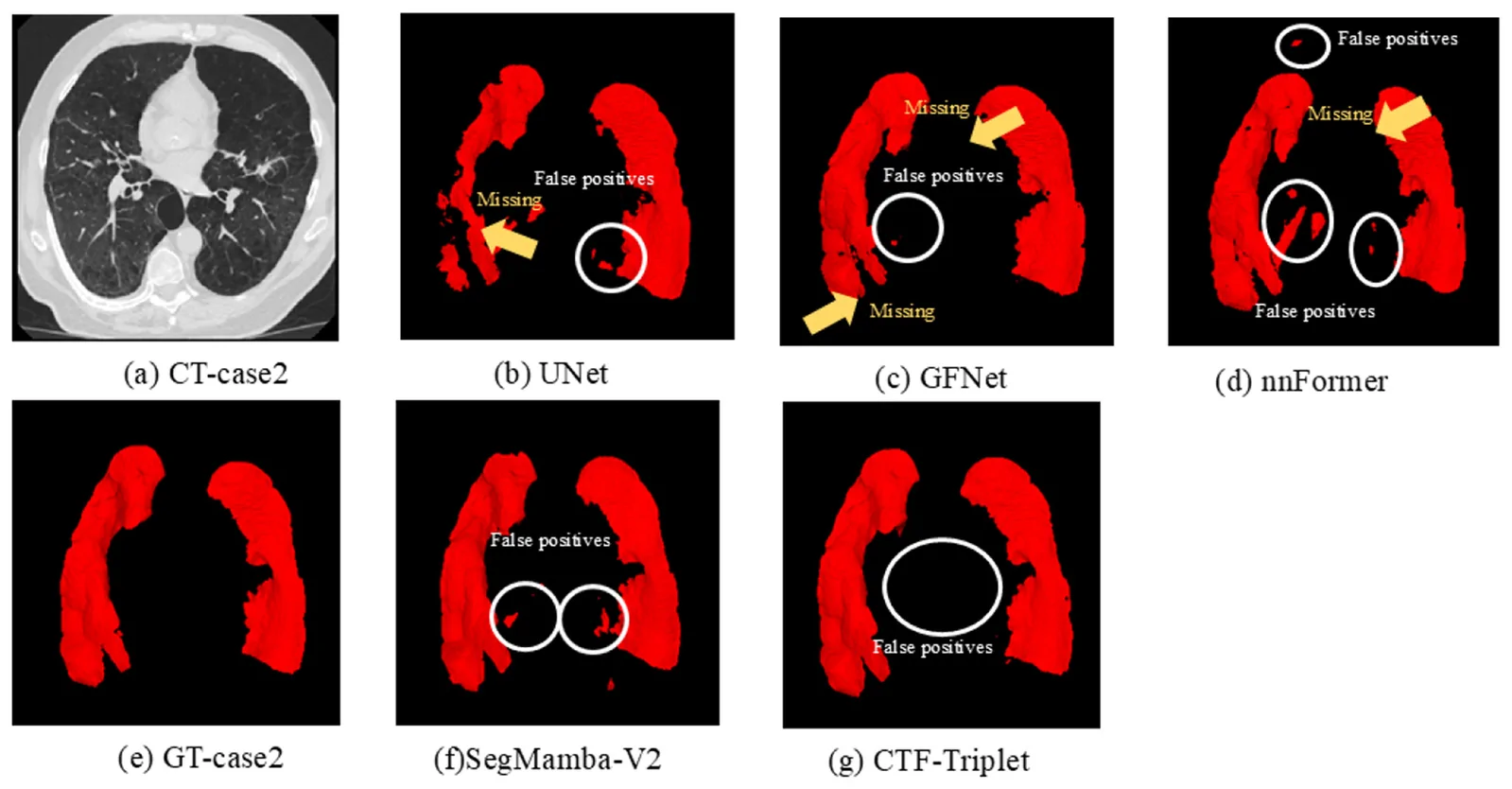
Visual comparisons of detection results generated by different models. **(a)** Coronal plane of the original 3D CT-2. **(b)** Ground truth (manual annotation). **(b–f)** Visualization of results from comparison models (UNet, GFNet, nnFormer, GT-case2, SegMamba-V2). **(g)** Detection result of the proposed CFT-Triplet model. The 3D views are provided for each result.

**Figure 5:**
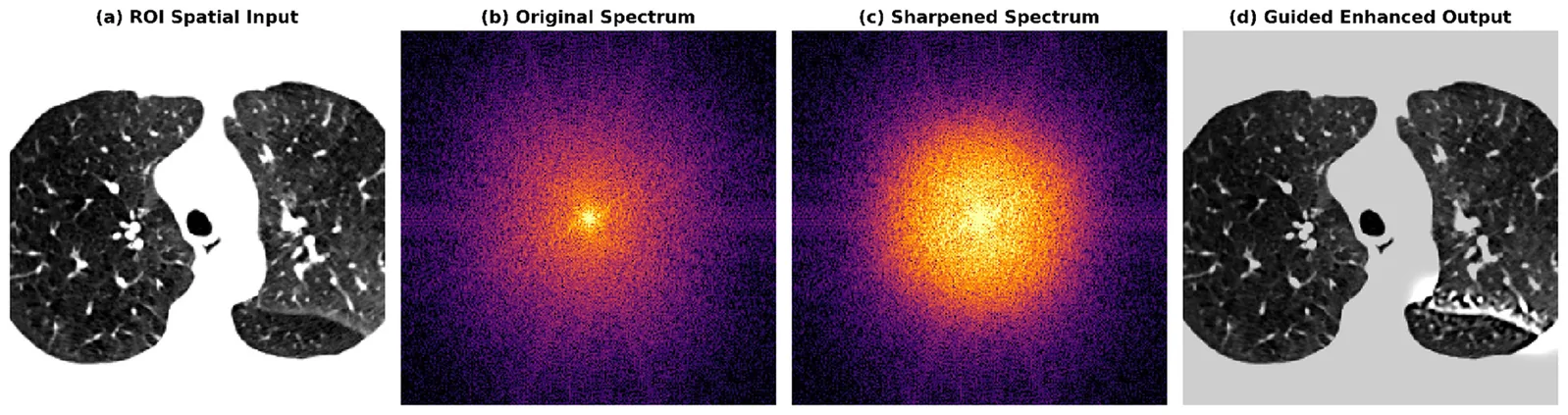
Visualized spectral validation of the frequency branch. **(a)** ROI Spatial Input. **(b)** Original Spectrum, where high frequencies are weak. **(c)** Sharpened Spectrum demonstrating targeted bandpass amplification. **(d)** Guided Enhanced Output explicitly verifying the Gate-Net Fusion, selectively sharpening fissures while suppressing background noise.

### 3.5 Generalizability and data heterogeneity

A persistent challenge in pulmonary fissure segmentation is the absence of large-scale, publicly available datasets with precise 3D voxel-level annotations. The extreme thinness of fissures makes manual delineation highly labor-intensive, rendering public benchmarks largely unavailable for this specific task. Pioneering works in this domain, such as FissureNet (Gerard et al., 2018), rely on private clinical cohorts. The lack of open-source implementations makes direct algorithmic benchmarking unfeasible. To evaluate model generalizability, we constructed the SPCH dataset to incorporate multi-source variability.

Based on our data screening records, the 400 enrolled cases were acquired using scanners from multiple vendors, including Siemens, Philips, and GE Medical Systems. The imaging protocols vary significantly, with slice thicknesses ranging from 0.625 mm to 1.25 mm and tube voltages including 100, 110, and 120 kVp. Additionally, the dataset incorporates diverse reconstruction kernels (e.g., LUNG, STANDARD, B35s, and Bl57d) and covers a wide demographic distribution (including cases originating from Germany, Spain, Italy, Austria, India, and China). This multi-vendor and multi-protocol diversity exposes the model to domain shifts during training and testing. The robust performance on our test set indicates that CFT-Triplet is not overfitted to a narrow institutional protocol, but adapts to different scanner characteristics and image qualities.

### 3.6 Computational efficiency

To quantify the efficiency of the Bidirectional Mamba2 architecture, we evaluated the computational complexity using the THOP profiling tool under a standard 96 × 96 × 96 input volume. Additionally, to provide an evaluation for clinical deployment, we measured the GPU memory consumption and inference time per volume on a single NVIDIA A100 GPU. Table 3 summarizes these metrics, demonstrating the linear complexity advantage of our method. CFT-Triplet uses 145.25 M parameters to capture fine-grained textures and spectral boundaries, requiring 221.70 G FLOPs, with an inference time of 76 ms and memory footprint of 9.4 GB. This overhead is lower than Transformer-based baselines like Swin UNETR (328.00 G, 112 ms) and nnFormer (240.20 G, 135 ms).

**Table 3:** Comparisons of computational efficiency (FLOPs, Parameters, Inference Time, and GPU Memory) under a standard 96 × 96 × 96 input volume.

Method	Architecture type	Params (M) ↓	FLOPs (G) ↓	Time (ms) ↓	Memory (GB) ↓
3D U-Net	CNN	19.00	400.00	58	7.2
Swin UNETR	Transformer	62.18	328.00	112	12.5
nnFormer	Transformer	149.30	240.20	135	14.8
**CFT-Triplet (Ours)**	**SSM+CNN**	**145.25**	**221.70**	**76**	**9.4**

Bold values indicate the best results.

### 3.7 Spectral validation and explainability

We validated the spectral sharpening effect by applying a 2D Fast Fourier Transform (FFT) to the feature maps extracted from the ROI spatial input (Figure 4a) before and after the Frequency Branch. The original spectrum (Figure 4b) indicates that high-frequency components corresponding to thin, low-contrast pulmonary fissures are weak and prone to oversmoothing. Figure 4c shows the spectrum after Frequency Branch processing. The module functions as an adaptive bandpass filter, amplifying mid-to-high frequency bands without introducing extreme-high-frequency noise. Figure 4d illustrates the effect of the Gate-Net Fusion Layer. Instead of a global high-pass filter, the network uses spatial context as a soft attention mask to guide spectral enhancement, suppressing background noise and sharpening fissure structures.

In the spatial domain, Figure 6 provides color overlays (Red for Ground Truth, Lime for CFT-Triplet Prediction, Yellow for Overlap). The visual alignment confirms that frequency-enhanced features improve topological continuity and boundary adherence.

**Figure 6:**
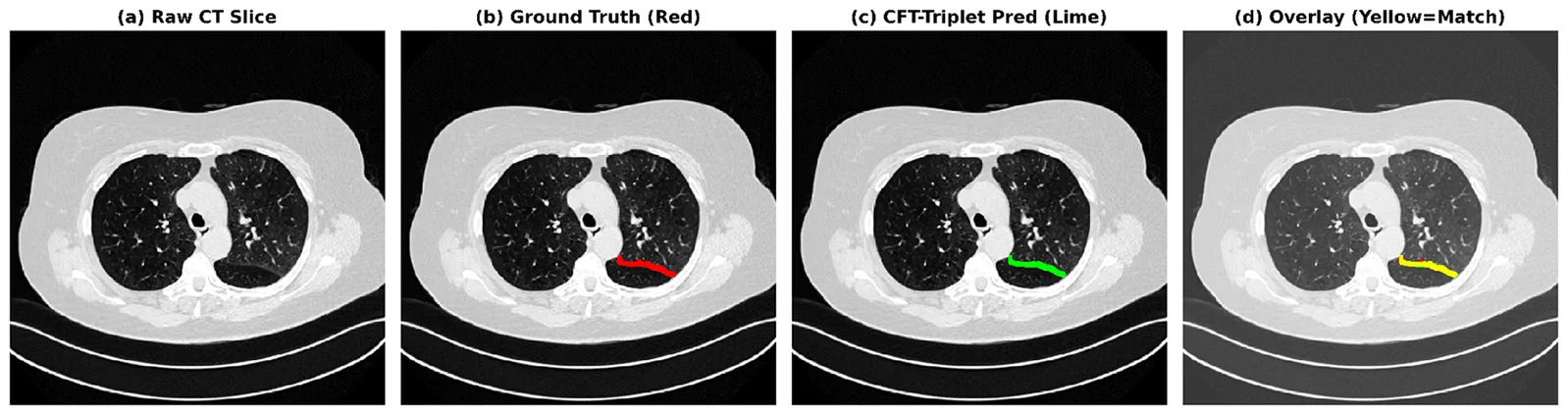
Visual comparisons of fissure detection results with quantitative overlays. **(a)** Raw CT slice. **(b)** Ground Truth annotated in Red. **(c)** CFT-Triplet prediction annotated in Lime. **(d)** Overlay showing the exact Match in Yellow. The visual alignment confirms the topological integrity and boundary adherence of our framework.

### 3.8 Failure case analysis

Despite its general robustness, CFT-Triplet occasionally struggles in extreme clinical conditions. In cases of severe emphysema, where lung parenchyma is heavily destructed and physical fissure boundaries are absent, the model has difficulty inferring the exact anatomical separation. As shown in Figure 7, the network relies on the contextual branch to estimate missing boundaries in these regions, leading to minor geometric deviations from manually annotated pseudo-boundaries. Future work will explore integrating prior anatomical shape models to regulate predictions in boundary-absent pathological regions.

**Figure 7:**
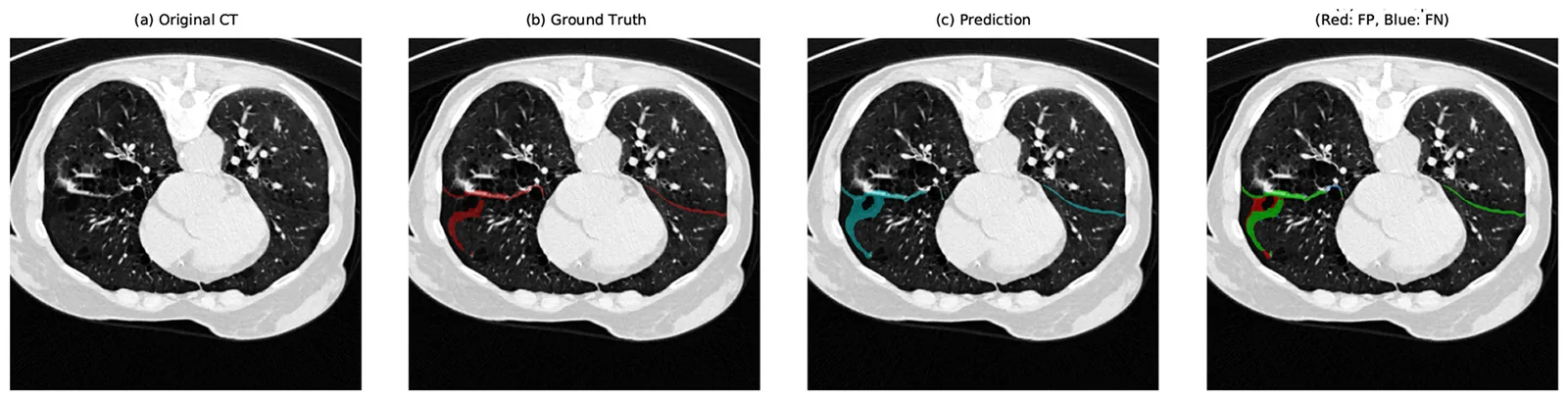
Failure case analysis of the CFT-Triplet model on a severe emphysema scan. **(a)** The original CT slice shows heavily destructed lung parenchyma where physical fissure boundaries are completely absent. **(b)** Ground truth annotation. **(c)** The prediction generated by CFT-Triplet. **(d)** The error map highlighting the geometric deviations, where green represents true positives, red indicates false positives (over-estimation), and blue indicates false negatives (under-estimation).

### 3.9 Ablation study

To verify the contribution of each key component in our proposed CFT-Triplet framework, we conducted a stepwise ablation study. We established a baseline using only the Texture Branch (a standard CNN encoder-decoder). Subsequently, we progressively integrated the Contextual Branch (Mamba2), the Frequency Branch, and the MoE Fusion module. Specifically, to justify the serial arrangement of the Contextual and Frequency branches, we compared three topological configurations: (1) *Frequency-First* (Frequency → Context); (2) *Parallel* (Frequency ∥ Context); and (3) *Serial* (Context → Frequency, our proposed design).

Table 4 summarizes the quantitative results of these configurations:

1. Baseline (texture only): Relies solely on local convolutions. Lacking global context modeling, it exhibits significant topological defects, yielding a high BNE of 1.650. This confirms that CNN architectures struggle to maintain fissure continuity.
2. + Context (Mamba2): Integrating the bidirectional Mamba2 branch mitigates this issue. The BNE drops to 0.450, indicating that global context modeling aids in repairing topological breaks.
3. + Frequency: The proposed Serial topology outperforms the Parallel and Frequency-First configurations, particularly in NSD (89.50% vs. 88.40% and 87.80%; Table 4). This supports the hypothesis that Mamba2's state space modeling attenuates high-frequency signals (spectral bias). Placing the Frequency Branch in series immediately after Mamba2 compensates for this loss, sharpening boundary details more effectively than parallel or reverse arrangements.
4. + MoE fusion (full model): Finally, incorporating the Scale-aware Mixture of Experts (MoE) provides the final refinement, pushing the DSC to 88.62% and reducing BNE to 0.300.

**Table 4:** Ablation study of key components and topological arrangements in the proposed CFT-Triplet.

Configurations	DSC (%) ↑	Pre (%) ↑	NSD (%) ↑	BNE ↓
Baseline (texture only)	80.45 ± 3.12	79.71 ± 3.25	81.20 ± 4.10	1.650 ± 0.850
+ Context (Mamba2)	85.15 ± 2.65	83.93 ± 2.85	86.50 ± 3.20	0.450 ± 0.220
+ Context + Freq (frequency-first)	86.20 ± 2.45	85.10 ± 2.35	87.80 ± 2.80	0.410 ± 0.190
+ Context + Freq (parallel)	86.85 ± 2.38	85.95 ± 2.20	88.40 ± 2.65	0.380 ± 0.180
+ Context + Freq (serial, ours)	87.50 ± 2.30	86.66 ± 2.10	89.50 ± 2.50	0.350 ± 0.150
**CFT-Triplet (+ MoE)**	88.62 ± 2.19	88.18 ± 2.05	92.15 ± 1.85	0.300 ± 0.150

Context: Mamba2-based Contextual Branch. Freq, Frequency Branch; MoE, Scale-aware Mixture of Experts Fusion. Bold values indicate the best results.

## 4 Conclusions

Pulmonary fissure detection plays a pivotal role in assessing lung structure and function, particularly for patients with chronic obstructive pulmonary disease (COPD) undergoing bronchoscopic lung volume reduction (BLVR). Traditional methods, which rely on handcrafted features or convolutional networks, struggle to capture the global continuity of weak fissures or discern the fine boundaries of abnormal pathologies.

In this study, we introduced CFT-Triplet, a hybrid framework engineered to address the challenges of pulmonary fissure segmentation. CFT-Triplet incorporates a Contextual Branch based on the Mamba2 model to capture long-range dependencies, aiding in repairing discontinuous weak fissures. A Frequency Branch is arranged in series to perform spectral sharpening, which suppresses background noise and refines boundaries. Furthermore, a Scale-aware Mixture of Experts (MoE) mechanism is deployed in the decoding stage to fuse multi-scale features, enhancing the model's adaptability to fissures of varying sizes and shapes.

We evaluated CFT-Triplet on a clinical dataset comprising 3D CT scans from 400 participants at the Shandong Provincial Chest Hospital (SPCH). Experimental results demonstrate that our framework outperforms CNN and Transformer-based methods. Compared to recent Transformer baselines (e.g., Swin UNETR, nnFormer) and State Space Models (U-Mamba, SegMamba-V2), CFT-Triplet achieves an increase in Dice Similarity Coefficient (DSC) ranging from 3.04% to 13.79%. Ablation studies confirmed that the integration of Mamba2 and frequency-domain filtering addresses the performance bottlenecks of traditional encoders.

In conclusion, CFT-Triplet offers a specialized solution for pulmonary fissure detection. By integrating texture, context, and spectral information, it yields quantitative improvements in boundary adherence and topological continuity, which may assist in the preoperative assessment and treatment planning of COPD patients.

## Data Availability

The original contributions presented in the study are included in the article/Supplementary material, further inquiries can be directed to the corresponding author.
