# Supplementary material for: CFT-Triplet: integrating contextual, frequency and textural features for fine-grained pulmonary fissure detection
Source: Front Artif Intell. 2026 Oct 20;9:1817367. doi: 10.3389/frai.2026.1817367 (PMC13727603; doi:10.3389/frai.2026.1817367)
Supplement: Supplementary file 1 [file Supplementary_file_1.pdf]

SUPPLEMENTARY DATA

To ensure absolute transparency and facilitate reproducibility, we provide the comprehensive hyperparameter settings and tuning protocols used during the training phase. All baseline models (CNN-based, Transformer-based, and SSM-based) and the proposed CFT-Triplet were trained on the identical Shandong Provincial Chest Hospital (SPCH) dataset split using the same hardware infrastructure (NVIDIA A100 GPUs).

The patch size for all 3D volumetric models was standardized to $160 \times 160 \times 160$ to maintain uniform spatial context during the extraction process. The specific optimizer configurations, learning rate schedules, and loss functions were optimized per architecture family to yield their respective best performances without penalizing the baselines.

Table 6. Supplementary Table S1. Detailed hyperparameter configurations and training protocols for the proposed CFT-Triplet and all evaluated baseline models.

Method	Optimizer	Initial LR	LR Scheduler	Batch Size	Epochs	Loss Function
U-Net	Adam	1×10^{-3}	Step Decay ($\gamma = 0.5$)	8	300	Dice + CE
V-Net	Adam	1×10^{-3}	Step Decay ($\gamma = 0.5$)	8	300	Dice + CE
Attention U-Net	Adam	1×10^{-3}	Step Decay ($\gamma = 0.5$)	8	300	Dice + CE
UNETR	AdamW	1×10^{-4}	Cosine Annealing	4	500	Dice + CE
TransBTS	AdamW	1×10^{-4}	Cosine Annealing	4	500	Dice + CE
Swin UNETR	AdamW	1×10^{-4}	Cosine Annealing	4	500	Dice + CE
nnFormer	SGD	1×10^{-2}	Poly Decay ($power = 0.9$)	4	500	Dice + CE
GFNet	AdamW	2×10^{-4}	Cosine Annealing	8	400	Dice + CE
U-Mamba	AdamW	1×10^{-4}	Cosine Annealing	8	400	Dice + CE
SegMamba-V2	AdamW	1×10^{-4}	Cosine Annealing	8	400	Dice + CE
**CFT-Triplet (Ours)**	**AdamW**	$\mathbf{1 \times 10^{-4}}$	**Supervised Decay** ($\gamma = 0.3$)	**8**	**400**	**Tversky + WBCE**
